# Supplementary material for: The dynamic landscape of transcription initiation in yeast mitochondria
Source: Nat Commun. 2020 Aug 27;11:4281. doi: 10.1038/s41467-020-17793-2 (PMC7452894; doi:10.1038/s41467-020-17793-2)
Supplement: Supplementary file 1 — Supplementary Information [file 41467_2020_17793_MOESM1_ESM.pdf]

The Dynamic Landscape of Transcription Initiation in Yeast Mitochondria

Sohn et al.

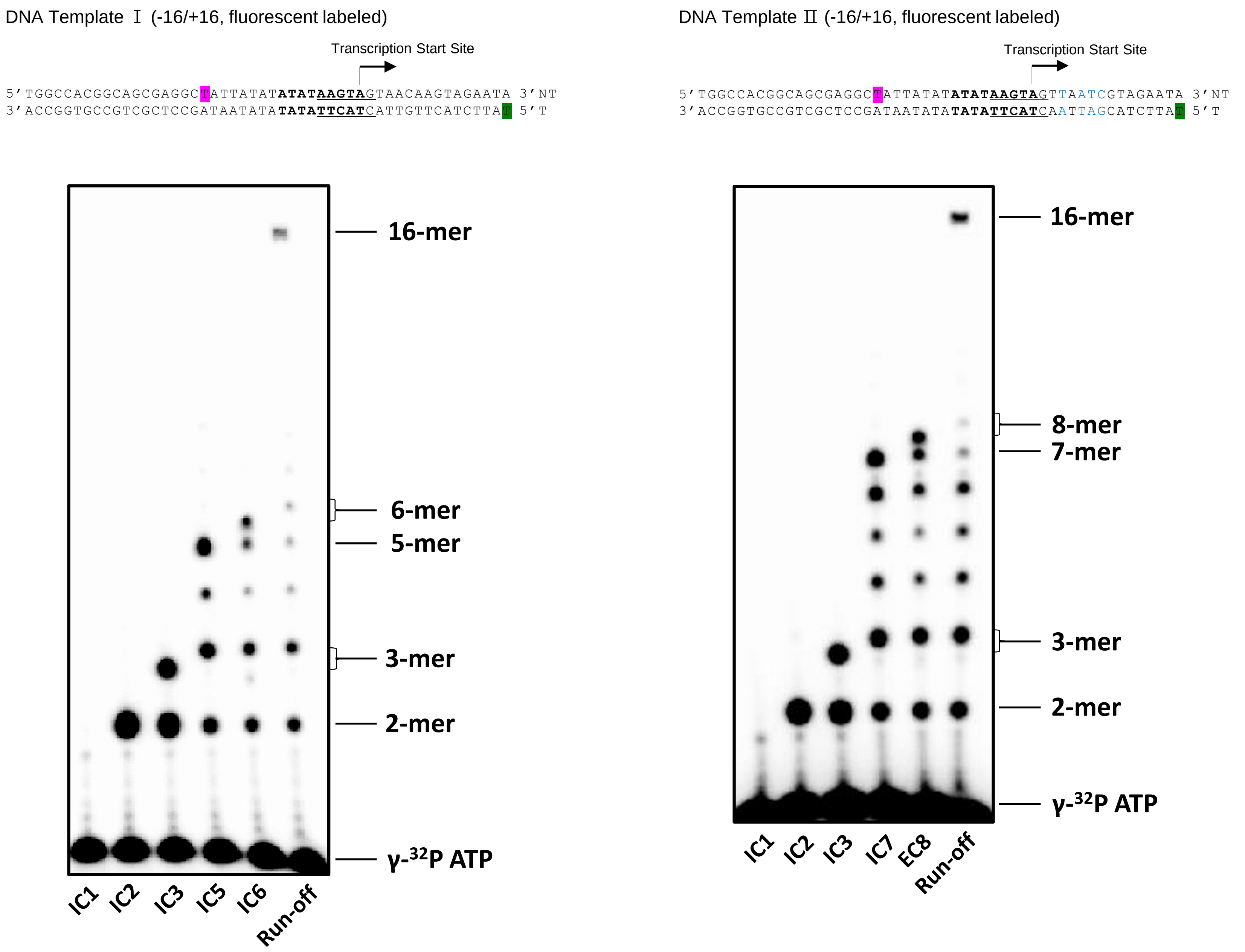

Supplementary Figure 1. Gel electrophoresis results from *in vitro* transcription assays performed on fluorescently labeled DNA templates I and II, showing abortive transcripts and full-length run-off products. Shifted bands within the same nucleotide length (3-mer, 6-mer, and 8-mer) are due to the difference between ribonucleotides and 3’-deoxyribonucleotides. The experiment was repeated three times showing similar results.

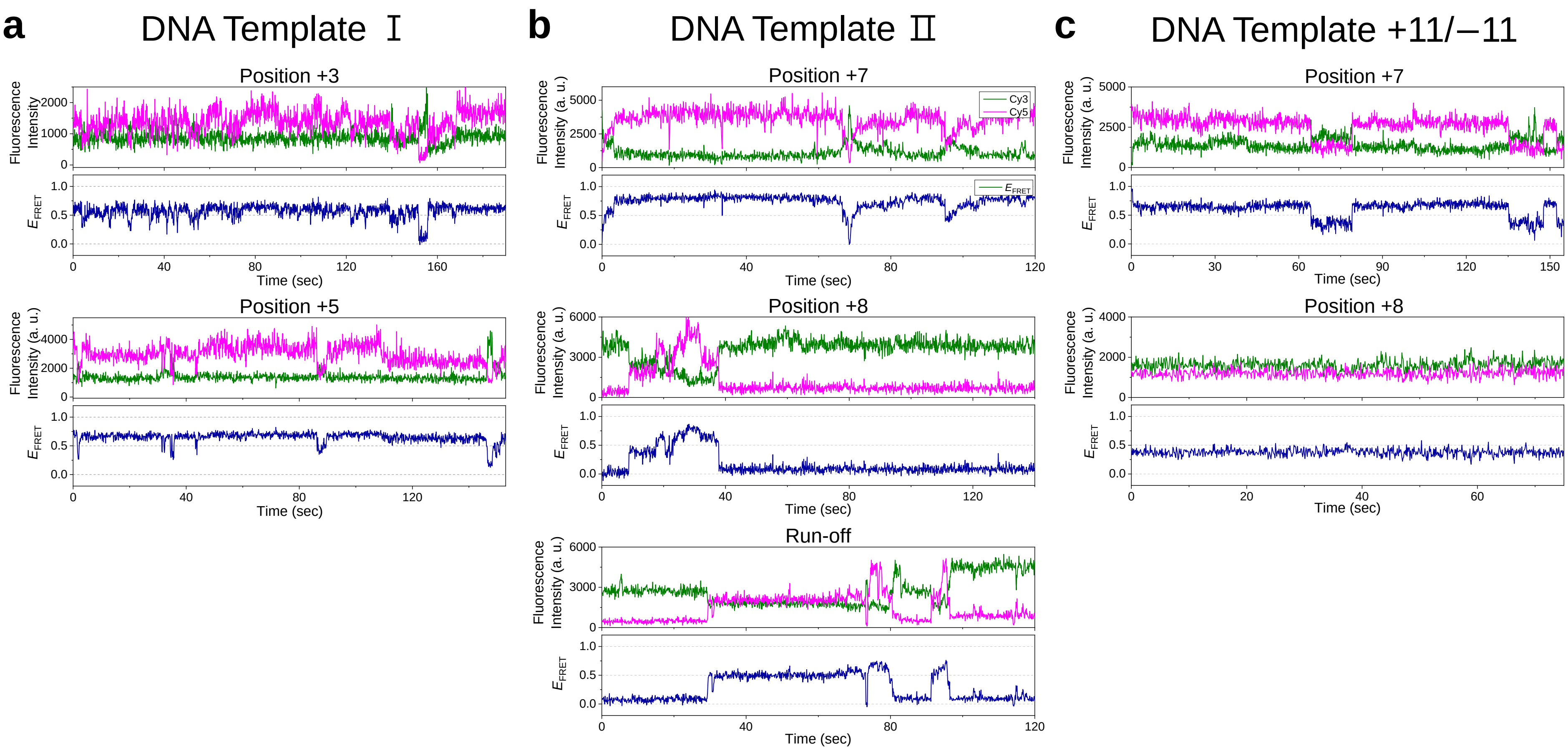

Supplementary Figure 2. a, Representative smFRET traces at positions +3 and +5 on DNA template Ⅰ. **b**, Representative smFRET traces at positions +7 and +8 and under run-off conditions on DNA template Ⅱ. **c**, Representative smFRET traces at positions +7 and +8 on DNA template +11/−11.

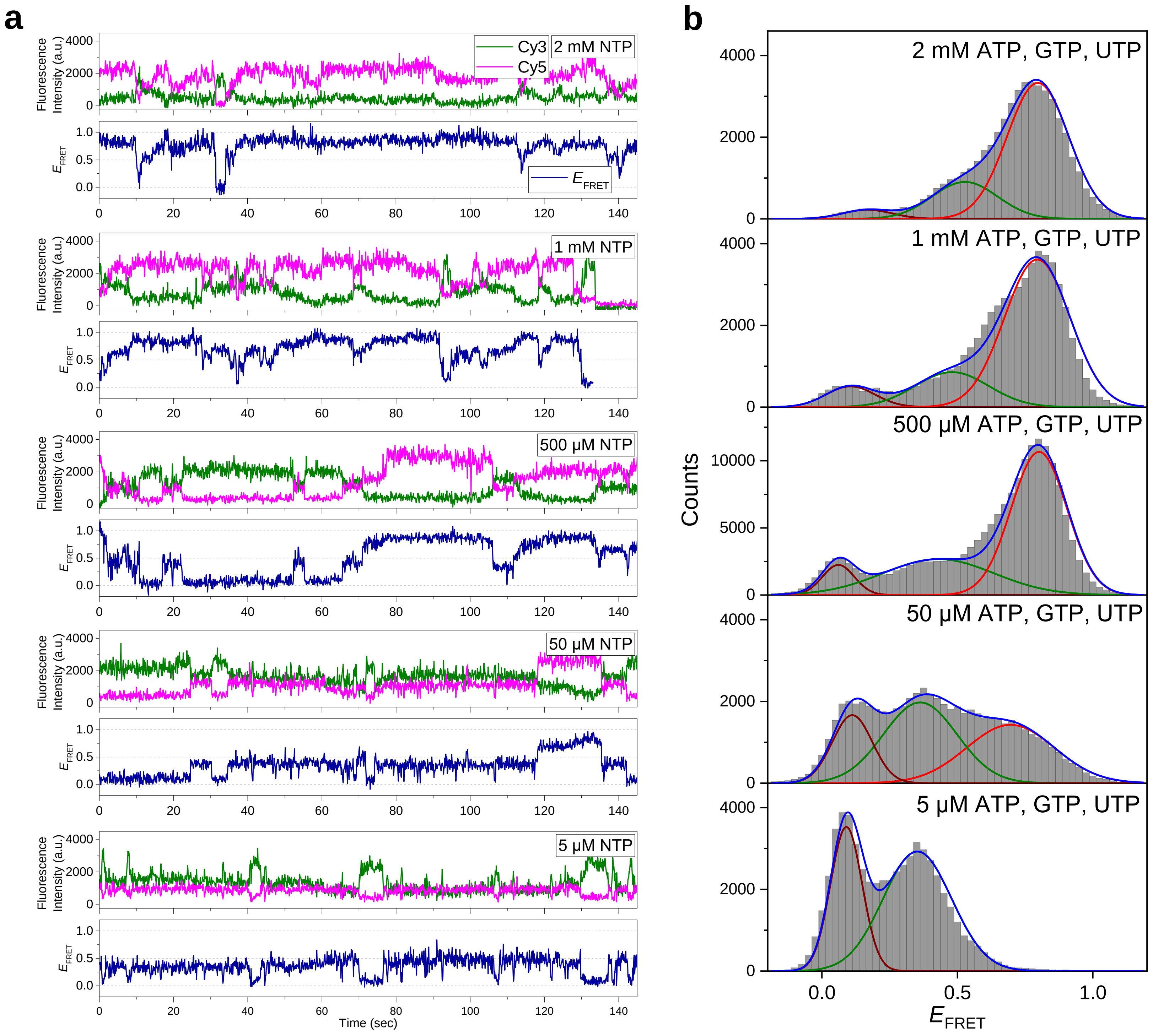

Supplementary Figure 3. a, Representative smFRET traces of the TIC using DNA template II at varying concentrations of ATP, GTP, UTP mix. The concentrations are those for each kind of nucleotide. **b**, FRET histograms obtained at each NTP concentration. 45, 51, 153, 46, and 45 traces were used for the histograms at 2 mM, 1 mM, 500 μM, 50 μM, and 5 μM NTP, respectively. Each histogram was fit to two or three Gaussian peaks as in Figure 1c (Supplementary Table 2).

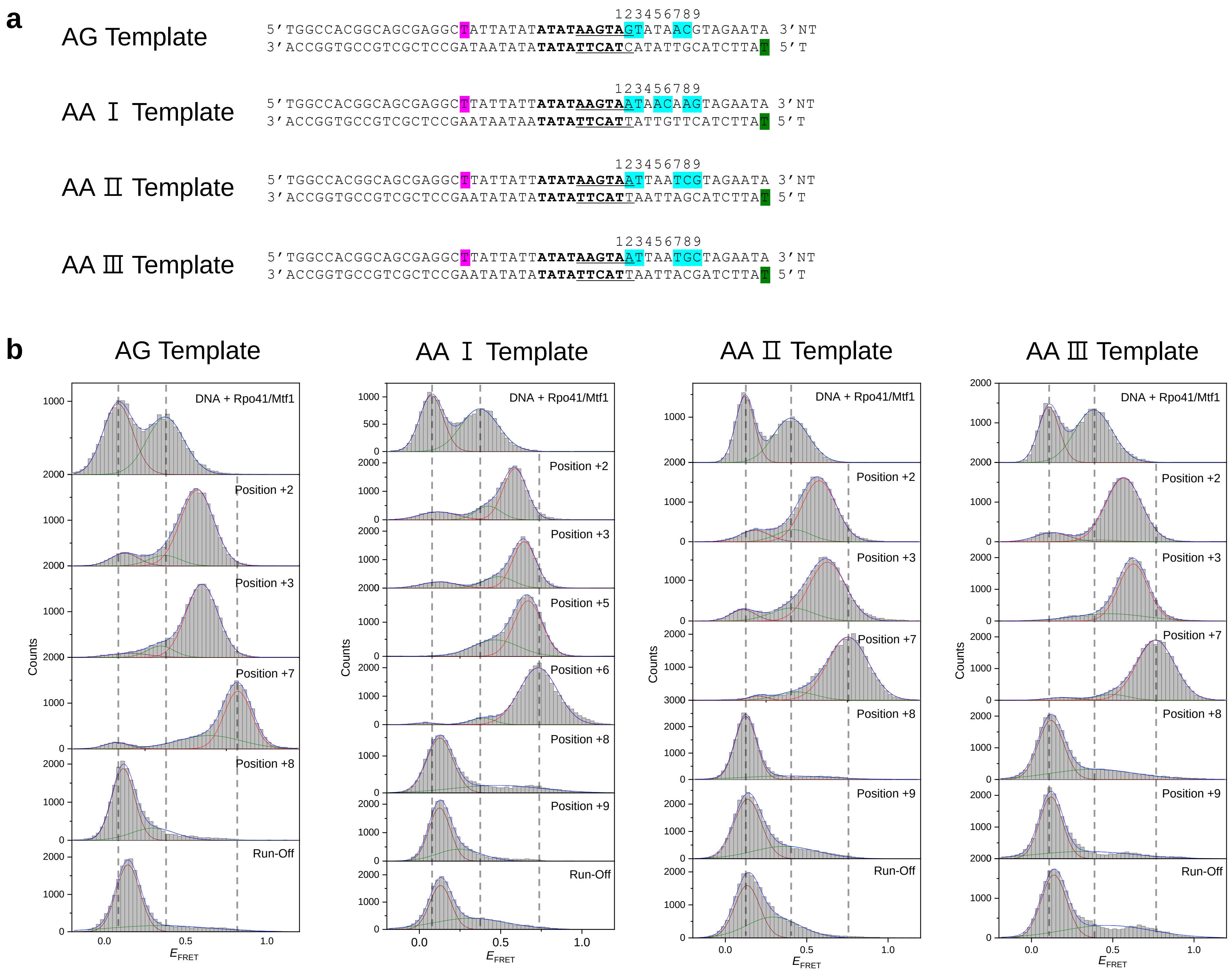

Supplementary Figure 4. a, Sequence designs of four different DNA templates having different nucleotide at position +2 (GTP or ATP) and different downstream sequences, shown in the same convention as in Figure 1b. intended stalling positions are highlighted in cyan. **b**, FRET histograms from single-molecule traces with colocalized Cy3 and Cy5 signals at each stalling position, shown along with fitting to single, double, or triple Gaussian peaks (Supplementary Table 2). Dashed vertical lines mark the major FRET peaks of DNA only, DNA + Rpo41/Mtf1, and IC7 (IC6 in case of AA I template).

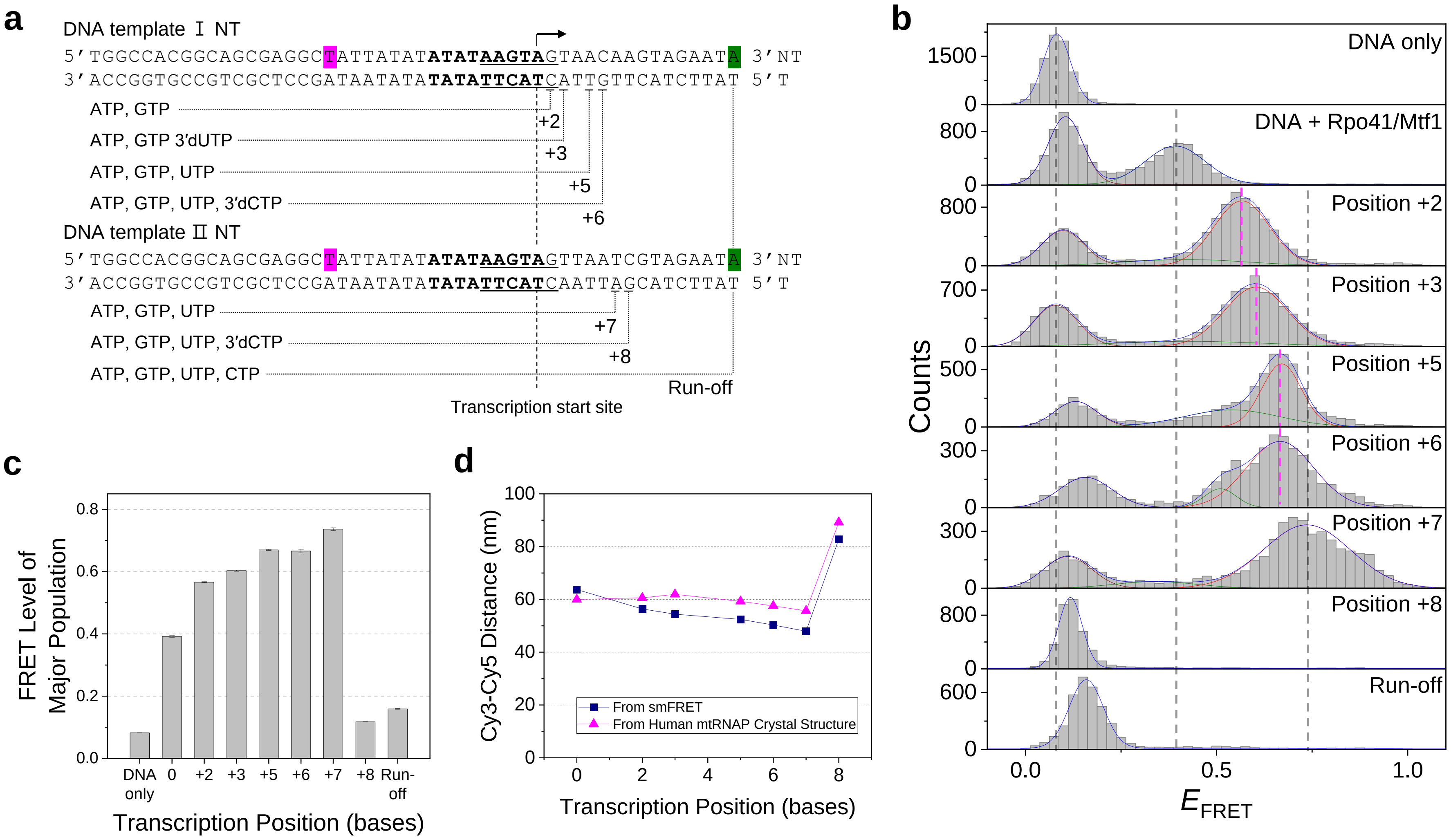

Supplementary Figure 5. a, Schematic design of the DNA templates used to measure the bending angles. The design is the same as that shown in Figure 1, with the exception that both Cy3 and Cy5 were located on the non-template strand at positions +16 and −16. **b**, FRET histograms shown with Gaussian fitting as described in Figure 1c (Supplementary Table 2). **c**, The FRET level of the major population in (**b**) shown for each stalling position as the center of the major Gaussian peak. The error bars represent the uncertainty in the peak center position from Gaussian fitting. **d**, Comparisons between the Cy3-Cy5 distances calculated from smFRET measurements and from the crystal structure of the human mitochondrial TIC (PDB: 6ERP).

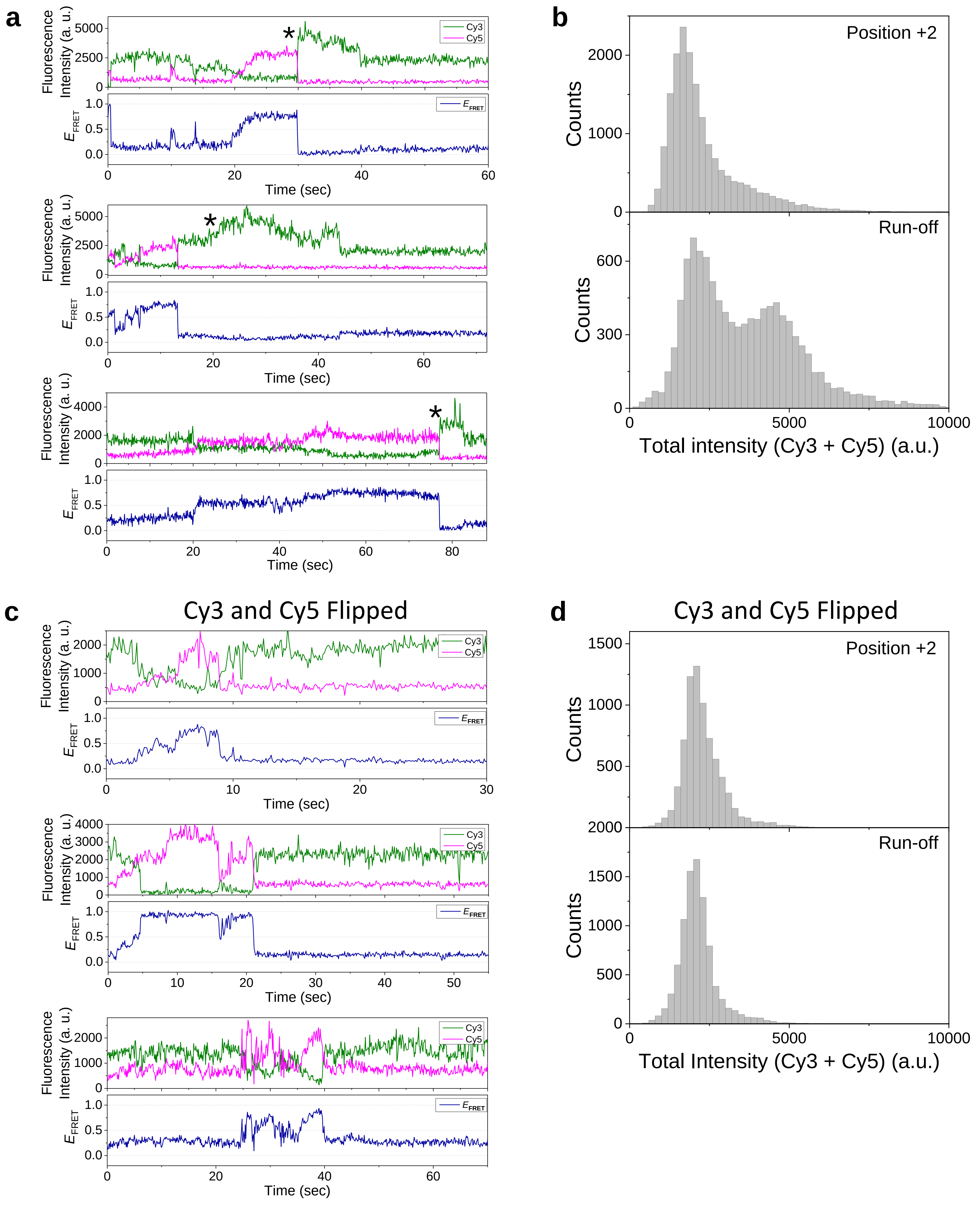

Supplementary Figure 6. a, Representative smFRET traces under run-off conditions for DNA template II, showing enhanced fluorescence signals (asterisks) following the drop in the FRET level. **b**, Histograms of total fluorescence intensities at position +2 and run-off conditions. **c**, Representative smFRET traces under run-off conditions for DNA template II with flipped Cy3/Cy5 labeling, showing no clear enhancement of fluorescence signals following the FRET level drop. **d**, Histograms of total fluorescence intensities at position +2 and run-off conditions obtained with flipped labeling.

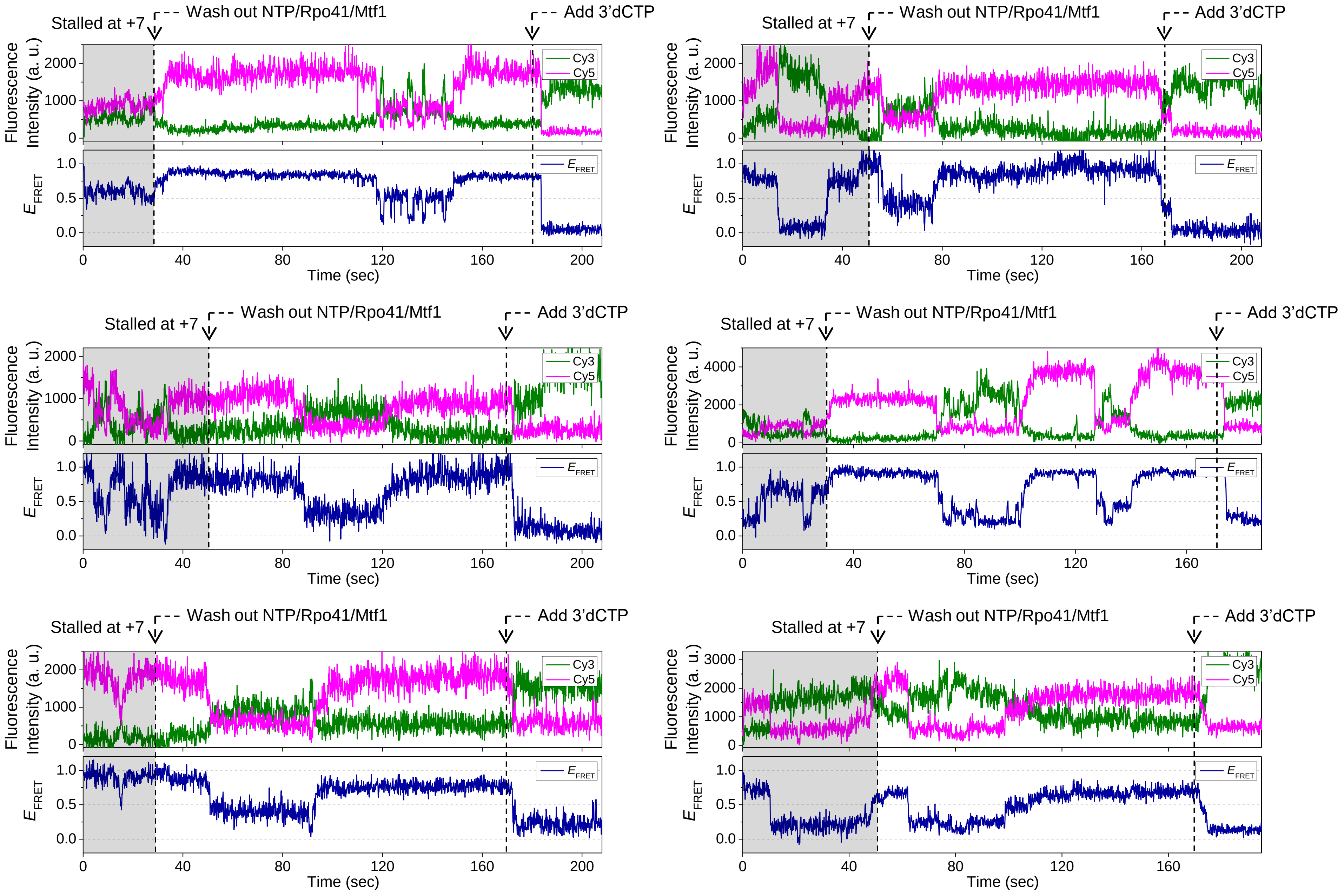

Supplementary Figure 7. Representative smFRET traces showing the conformational dynamics after washing out the NTP mix (first arrow) from the TIC of DNA template II equilibrated at position +7 (gray region; 0.5 mM each of ATP, GTP, and UTP). Subsequently, 0.5 mM 3'dCTP was added to promote progression to position +8 (second arrow). The abrupt drop in the FRET efficiency indicates successful progression to position +8.

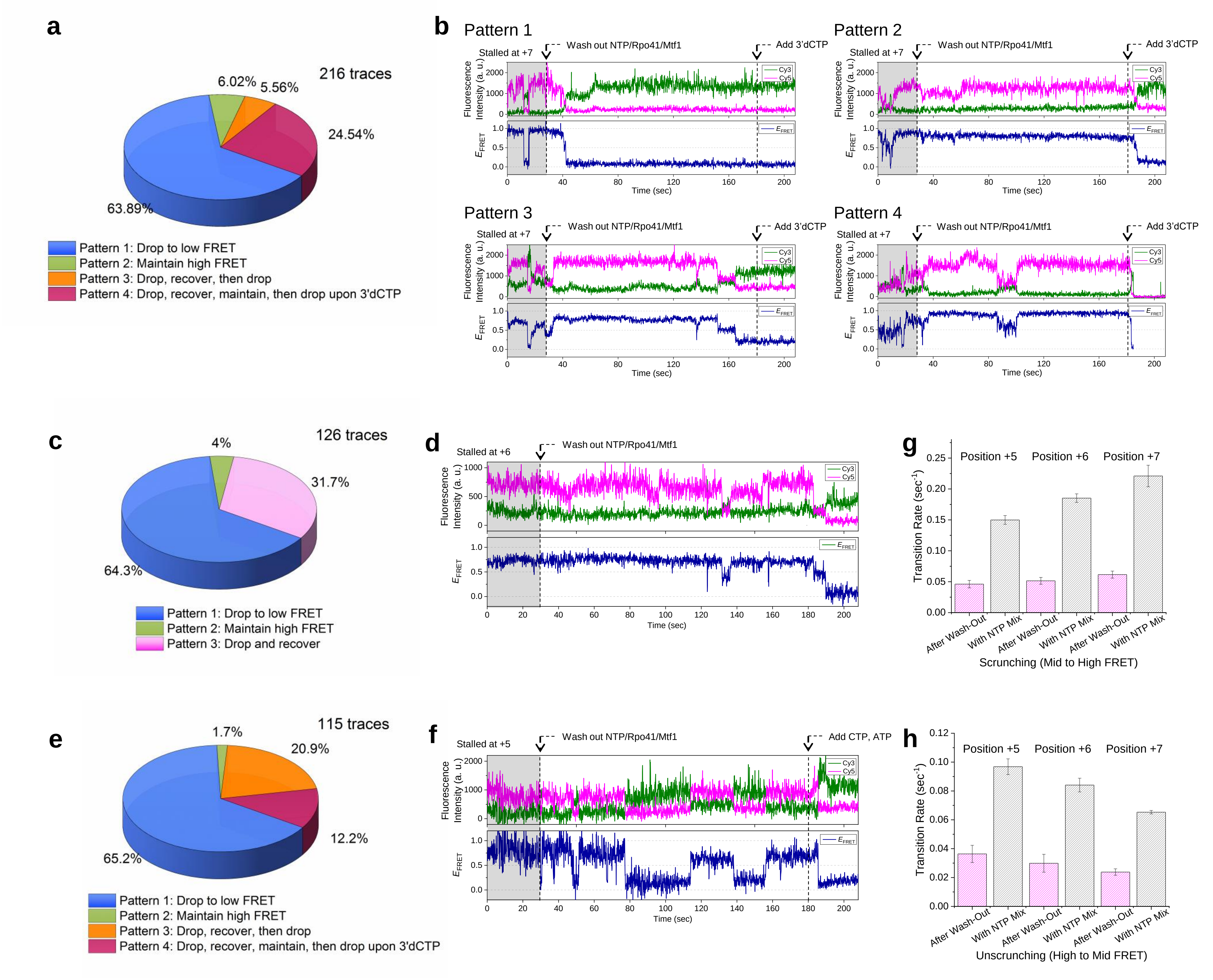

Supplementary Figure 8. a, Populations of four distinct patterns of single-molecule traces observed in NTP wash-out experiments at stalling position +7. Pattern 1: FRET level dropped and remained low after NTP wash-out. Pattern 2: FRET level remained at high FRET until flowing in 3'dCTP. Pattern 3: FRET level dropped and recovered back to high level, but dropped again before flowing-in 3'dCTP, preventing to check the capability of progressing to elongation. Pattern 4: FRET level dropped, recovered, and remained high until flowing-in 3'dCTP, showing the capability of progressing to elongation. Patterns 3 and 4 represent smFRET traces showing FRET level drop followed by the recovery of high FRET level. **b**, Example traces for each pattern of single-molecule trace. **c**, Populations of three distinct patterns of traces obtained from the same kind of experiment as in (**a**, **b**) at stalling position +6. Pattern 3 corresponds to the sum of patterns 3 and 4 in (**a**, **b**), which could not be distinguished because stalled IC6 does not allow further extension due to 3'dCTP. **d**, Example trace showing pattern 3, i.e. scrunching-unscrunching dynamics after NTP wash-out. **e**, **f**, Same as (**c**, **d**) but at stalling position +5. **g**, **h**, Scrunching (**g**) and unscrunching (**h**) rates compared between stalling positions in the presence or absence of NTP. Data represent mean and s.e.m. from three independent measurements.

Fig. 1c: DNA Template I and II Labeled at +16/-16

		DNA Only	Position 0	Position +2	Position +3	Position +5	Position +6	Position +7	Position +8	Run-Off
Low FRET	E_{FRET}	0.138±0.00036	0.135±0.00085	0.0974±0.0058	0.129±0.039	0.110±0.0064	0.0913±0.004	0.0697±0.0061	0.112±0.00081	0.145±0.0018
	Width	0.128±0.00072	0.12±0.0017	0.166±0.012	0.228±0.047	0.177±0.013	0.282±0.068	0.143±0.013	0.126±0.0019	0.155±0.0068
	Area	2690±13	2220±29	673±43	951±310	1210±81	436±91	432±42	2210±36	2890±290
Mid FRET	E_{FRET}	-	0.376±0.00082	0.402±0.018	0.414±0.014	0.446±0.012	0.553±0.014	0.56±0.026	0.702±0.029	0.345±0.045
	Width	-	0.164±0.0018	0.179±0.028	0.22±0.035	0.250±0.031	0.32±0.046	0.446±0.037	0.856±0.39	0.340±0.051
	Area	-	3610±33	1130±240	2750±600	2020±87	2050±92	1830±200	308±110	1390±320
High FRET	E_{FRET}	-	-	0.563±0.0024	0.630±0.0033	0.665±0.0017	0.771±0.0013	0.811±0.002	-	-
	Width	-	-	0.135±0.0025	0.191±0.0028	0.137±0.0032	0.131±0.0029	0.180±0.0066	-	-
	Area	-	-	4260±220	6620±300	3780±110	3340±100	2180±160	-	-

Fig. 2d: DNA Template +11/-11

		DNA Only	Position 0	Position +7	Position +8	Run-Off
Low FRET	E_{FRET}	0.269±0.00029	0.327±0.0031	0.360±0.014	0.318±0.019	0.279±0.0015
	Width	0.0895±0.00058	0.163±0.0062	0.246±0.03	0.131±0.027	0.151±0.0031
	Area	196±1.1	55.1±1.8	26.7±2.7	24.2±6.3	126±2.2
Mid FRET	E_{FRET}	-	0.753±0.0011	0.641±0.0019	0.442±0.0023	0.725±0.0059
	Width	-	0.151±0.0022	0.116±0.0038	0.0923±0.0032	0.154±0.012
	Area	-	141±1.8	124±4.8	87.2±6.2	34±2.2
High FRET	E_{FRET}	-	-	0.774±0.0061	0.715±0.0043	-
	Width	-	-	0.0949±0.0088	0.167±0.0091	-
	Area	-	-	28.5±3.9	38.6±1.8	-

Fig. 5a: NTP Wash-Out Experiment

**0 min**		Position +2	Position +7	Position +8 (+11/-11)	Run-Off	**3 min**	Position +2	Position +7	Position +8 (+11/-11)	Run-Off
Low FRET	E_{FRET}	0.119±0.0014	0.104±0.016	0.332±0.0044	0.137±0.00024		0.131±0.0015	0.132±0.0088	0.332±0.0044	0.136±0.0014
	Width	0.104±0.003	0.154±0.033	0.145±0.013	0.0661±0.00049		0.0959±0.0031	0.151±0.018	0.182±0.027	0.0814±0.0029
	Area	60.5±1.7	17.4±3.2	27.3±2.5	210±1.6		17.6±0.53	9.18±0.94	4.94±0.72	24.9±0.87
Mid FRET	E_{FRET}	0.447±0.02	0.408±0.0079	0.443±0.00091	0.229±0.0037		0.433±0.0018	0.408±0.0079	0.451±0.0016	0.395±0.0056
	Width	0.206±0.024	0.214±0.026	0.0908±0.0019	0.108±0.0078		0.157±0.0039	0.198±0.018	0.116±0.0031	0.218±0.012
	Area	80.9±15	15.1±3	83.2±1.7	32.8±2.2		30.1±0.73	17.6±0.91	20±0.51	28.3±1.3
High FRET	E_{FRET}	0.626±0.0047	0.849±0.0017	0.715±0.0043	-		-	0.845±0.0034	0.733±0.0057	-
	Width	0.155±0.0051	0.184±0.0034	0.164±0.0095	-		-	0.196±0.0068	0.148±0.012	-
	Area	152±14	214±3.4	37.4±2.1	-		-	34.8±1	5.88±0.48	-
**1 min**						**4 min**				
Low FRET	E_{FRET}	0.138±0.0021	0.122±0.0096	0.332±0.0044	0.14±0.00037		0.131±0.002	0.115±0.0068	0.332±0.0044	0.136±0.0016
	Width	0.105±0.0045	0.155±0.019	0.122±0.016	0.0666±0.00073		0.0924±0.0041	0.130±0.014	0.098±0.017	0.0908±0.0033
	Area	15.8±0.63	7.09±0.76	4.88±0.65	32.5±0.38		18.9±0.77	9.05±0.82	3.66±0.62	17.8±0.56
Mid FRET	E_{FRET}	0.441±0.0019	0.408±0.0079	0.446±0.0013	0.247±0.0052		0.438±0.0021	0.408±0.0079	0.450±0.0018	0.422±0.0019
	Width	0.144±0.004	0.216±0.022	0.0955±0.0027	0.109±0.0076		0.145±0.0044	0.204±0.011	0.083±0.0033	0.144±0.0038
	Area	28.7±0.78	9.61±0.73	17.8±0.49	8.01±0.51		34.7±1.0	18.8±0.84	12.4±0.49	31.0±0.7
High FRET	E_{FRET}	-	0.828±0.0026	0.733±0.0088	-		-	0.853±0.0038	0.745±0.0046	-
	Width	-	0.214±0.0053	0.13±0.019	-		-	0.197±0.0076	0.0761±0.0096	-
	Area	-	41±0.88	3.6±0.51	-		-	30.3±1	3.49±0.41	-
**2 min**						**5 min**				
Low FRET	E_{FRET}	0.132±0.0023	0.132±0.0098	0.332±0.0044	0.138±0.00069		0.124±0.0015	0.118±0.0055	0.332±0.0044	0.128±0.0017
	Width	0.0929±0.0048	0.159±0.02	0.137±0.015	0.0793±0.0019		0.0942±0.0031	0.144±0.011	0.106±0.014	0.0841±0.0035
	Area	17.3±0.83	8.31±0.9	3.97±0.45	33.8±1.1		16±0.49	13.2±0.88	4.62±0.59	16.7±0.59
Mid FRET	E_{FRET}	0.436±0.0025	0.408±0.0079	0.452±0.00097	0.334±0.013		0.421±0.0016	0.408±0.0079	0.449±0.0017	0.418±0.0028
	Width	0.151±0.0054	0.192±0.012	0.0944±0.002	0.297±0.023		0.148±0.0034	0.213±0.0076	0.0873±0.0031	0.158±0.0056
	Area	33±1.1	13.7±0.85	15±0.32	21.7±1.7		28.7±0.65	26.8±0.86	12.8±0.46	26.5±0.81
High FRET	E_{FRET}	-	0.835±0.0025	0.733±0.0075	-		-	0.841±0.0035	0.742±0.0031	-
	Width	-	0.188±0.0051	0.164±0.017	-		-	0.195±0.007	0.101±0.0065	-
	Area	-	40.4±0.94	3.99±0.4	-		-	32.9±1	7.23±0.44	-

Supplementary Table 1. Summary of multi-peak Gaussian fitting to the FRET histograms in main figures, showing the respective peak position (E_{FRET}), peak width, and area under the Gaussian curve for low, mid and high FRET states.

Sup. Fig. 3b: NTP Concentration Dependence at Position +7

		2 mM	1 mM	500 uM	50 uM	5 uM
Low FRET	E_{FRET}	0.168±0.013	0.106±0.026	0.0608±0.0078	0.696±0.019	0.0902±0.0013
	Width	0.182±0.025	0.180±0.051	0.118±0.021	0.324±0.021	0.117±0.0026
	Area	50.9±6.8	115±33	332±83	580±66	515±14
Mid FRET	E_{FRET}	0.528±0.012	0.481±0.015	0.429±0.012	0.363±0.0079	0.352±0.0027
	Width	0.241±0.021	0.271±0.062	0.411±0.069	0.271±0.024	0.253±0.0057
	Area	273±32	291±62	1370±200	671±88	928±17
High FRET	E_{FRET}	0.797±0.0034	0.795±0.0046	0.802±0.0024	0.112±0.0035	-
	Width	0.228±0.0038	0.243±0.0072	0.206±0.0048	0.148±0.0062	-
	Area	950±29	1100±41	2750±110	308±30	-

Sup. Fig. 4b: AG, AA I, AA II, AA III Templates

**AG Template**		Position 0	Position +2	Position +3	Position +5	Position +6	Position +7	Position +8	Position +9	Run-Off
Low FRET	E_{FRET}	0.0834±0.0023	0.124±0.012	0.150±0.0081	-	-	0.0744±0.0059	0.116±0.0013	-	0.145±0.00091
	Width	0.184±0.0041	0.173±0.017	0.246±0.031	-	-	0.153±0.012	0.140±0.0024	-	0.146±0.0023
	Area	223±5.5	60.2±8.7	27.8± 3.2	-	-	25.8±1.8	329±6.1	-	329±6.6
Mid FRET	E_{FRET}	0.375±0.0033	0.37±0.0084	0.345±0.0064	-	-	0.645±0.014	0.308±0.0012	-	0.344±0.029
	Width	0.229±0.0062	0.195±0.047	0.166±0.014	-	-	0.395±0.014	0.267±0.023	-	0.605±0.044
	Area	224±5.8	56.2±18	51.4±4.6	-	-	144±9.4	107±8.6	-	125±10
High FRET	E_{FRET}	-	0.571±0.0021	0.600±0.0012	-	-	0.822±0.00089	-	-	-
	Width	-	0.204±0.0022	0.197±0.0021	-	-	0.170±0.0027	-	-	-
	Area	-	425±10	395±4.3	-	-	269±8.4	-	-	-
**AA I Template**										
Low FRET	E_{FRET}	0.0741±0.0014	0.116±0.011	0.122±0.0070	-	0.0387 0.049	-	0.126±0.0013	0.124±0.0011	0.130±0.00068
	Width	0.145±0.0027	0.226±0.023	0.214±0.015	-	0.150±0.0082	-	0.162±0.0038	0.131±0.0037	0.131±0.0018
	Area	186±3.4	79.0± 96.9	58.6±3.5	-	11.7±5.3	-	301±11	306±20	263±5.0
Mid FRET	E_{FRET}	0.371±0.0023	0.424±0.020	0.488±0.025	0.469±0.023	0.389±0.014	-	0.479±0.038	0.245±0.021	0.314±0.0081
	Width	0.231±0.0049	0.156±0.030	0.186±0.031	0.283±0.026	0.149±0.030	-	0.542±0.057	0.269±0.018	0.450±0.010
	Area	226±4.0	93.5±28	92.9±25	171±25	43.5±7.6	-	138±15	141±21	229±7.0
High FRET	E_{FRET}	-	0.588±0.0053	0.645±0.0036	0.668±0.0020	0.732±0.0021	-	-	-	-
	Width	-	0.146±0.0056	0.142±0.0035	0.171±0.0038	0.235±0.0046	-	-	-	-
	Area	-	329±26	289±24	347±24	588±9.4	-	-	-	-
**AA II Template**										
Low FRET	E_{FRET}	0.121±0.00091	0.180±0.0044	0.108±0.0098	-	-	0.207±0.0025	0.125±0.00057	0.137±0.0013	0.134±0.0013
	Width	0.116 ± 0.0018	0.183±0.0092	0.153± 0.017	-	-	0.113±0.042	0.131±0.0015	0.167±0.0049	0.154±0.0047
	Area	211 ± 3.2	65.6±2.8	53.1±8.3	-	-	18.2±9.4	392±6.1	455±28	306±22
Mid FRET	E_{FRET}	0.402±0.0019	0.420±0.0065	0.417 ± 0.0046	-	-	0.440±0.0017	0.379±0.034	0.366±0.033	0.293±0.016
	Width	0.217±0.0041	0.214±0.0068	0.263±0.046	-	-	0.239±0.060	0.52378±0.049	0.395±0.039	0.321±0.015
	Area	265±4.1	80.0±0.15	106±16	-	-	74.9±19	87.8±9.3	223±32	254±24
High FRET	E_{FRET}	-	0.577±0.00087	0.627±0.0018	-	-	0.754±0.0028	-	-	-
	Width	-	0.206±0.0018	0.223±0.0040	-	-	0.249±0.0043	-	-	-
	Area	-	398±3.0	405±9.4	-	-	595±12	-	-	-
**AA III Template**										
Low FRET	E_{FRET}	0.107±0.0015	0.128±0.0086	-	-	-	0.189±0.034	0.120±0.00082	0.120±0.0014	0.139±0.0016
	Width	0.129±0.003	0.207±0.016	-	-	-	0.190±0.065	0.157±0.0024	0.141±0.0035	0.155±0.0047
	Area	226±5.6	58.6±4.6	-	-	-	20.2±6.7	367±8.6	345±11	309±14
Mid FRET	E_{FRET}	0.382±0.0022	0.402±0.012	0.481±0.021	-	-	0.498±0.031	0.379±0.017	0.329±0.037	0.465±0.029
	Width	0.228±0.0049	0.249±0.011	0.441±0.022	-	-	0.201±0.067	0.518±0.023	0.593±0.0536	0.539±0.046
	Area	380±6.7	13.7±13	126±12	-	-	45.2±20	210±12	171±18	214±20
High FRET	E_{FRET}	-	0.568±0.0022	0.626±0.00096	-	-	0.764±0.0039	-	-	-
	Width	-	0.218±0.0030	0.181±0.0026	-	-	0.233±0.0054	-	-	-
	Area	-	440±9.7	407±11	-	-	555±17	-	-	-

Sup. Fig. 5b: DNA Template I NT and II NT Labeled at +16/-16

		DNA Only	Position 0	Position +2	Position +3	Position +5	Position +6	Position +7	Position +8	Run-Off
Low FRET	E_{FRET}	0.0821±0.00028	0.105±0.0011	0.0981±0.002	0.0796±0.0022	0.131±0.0038	0.157±0.005	0.111±0.009	0.117±0.00064	0.159±0.00086
	Width	0.0714±0.00058	0.0908±0.0023	0.111±0.004	0.114±0.0045	0.109±0.0075	0.137±0.01	0.125±0.016	0.0621±0.0013	0.0887±0.0018
	Area	196±1.4	115±2.7	66.7±2.1	73.6±2.6	30.4±1.8	27.4±1.7	26.2±4.7	81.9±1.6	80.5±1.5
Mid FRET	E_{FRET}	-	0.392±0.0025	0.422±0.0041	0.429±0.0062	0.538±0.0081	0.511±0.0091	0.343±0.061	-	-
	Width	-	0.156±0.0054	0.318±0.0072	0.404±0.0042	0.251±0.0035	0.0829±0.019	0.213±0.16	-	-
	Area	-	112±3.8	34.4±4.8	31.4±6.3	46.7±3.1	10.4±3.9	9.39±6	-	-
High FRET	E_{FRET}	-	-	0.566±0.0014	0.603±0.002	0.67±0.0016	0.666±0.0058	0.736±0.0044	-	-
	Width	-	-	0.145±0.0029	0.164±0.0043	0.104±0.0033	0.175±0.011	0.228±0.0095	-	-
	Area	-	-	161±3.7	151±4.6	71.3±2.4	76.7±4.4	95.4±3.3	-	-

Supplementary Table 2. Summary of multi-peak Gaussian fitting to the FRET histograms in supplementary figures, showing the respective peak position (E_{FRET}), peak width, and area under the Gaussian curve for low, mid and high FRET states.
