# Supplementary material for: The dynamic landscape of transcription initiation in yeast mitochondria
Source: Nat Commun. 2020 Aug 27;11:4281. doi: 10.1038/s41467-020-17793-2 (PMC7452894; doi:10.1038/s41467-020-17793-2)
Supplement: Supplementary file 3 — Reporting Summary [file 41467_2020_17793_MOESM3_ESM.pdf]

nature research

Corresponding author(s): Hajin Kim

Last updated by author(s): Apr 15, 2020

Reporting Summary

Nature Research wishes to improve the reproducibility of the work that we publish. This form provides structure for consistency and transparency in reporting. For further information on Nature Research policies, see Authors & Referees and the Editorial Policy Checklist.

Statistics

For all statistical analyses, confirm that the following items are present in the figure legend, table legend, main text, or Methods section.

n/a	Confirmed	
☐	☒	The exact sample size (*n*) for each experimental group/condition, given as a discrete number and unit of measurement
☐	☒	A statement on whether measurements were taken from distinct samples or whether the same sample was measured repeatedly
✓	☒	The statistical test(s) used AND whether they are one- or two-sided<br>*Only common tests should be described solely by name; describe more complex techniques in the Methods section.*
✓	☒	A description of all covariates tested
✓	☒	A description of any assumptions or corrections, such as tests of normality and adjustment for multiple comparisons
☐	☒	A full description of the statistical parameters including central tendency (e.g. means) or other basic estimates (e.g. regression coefficient) AND variation (e.g. standard deviation) or associated estimates of uncertainty (e.g. confidence intervals)
✓	☒	For null hypothesis testing, the test statistic (e.g. *F*, *t*, *r*) with confidence intervals, effect sizes, degrees of freedom and *P* value noted<br>*Give P values as exact values whenever suitable.*
✓	☒	For Bayesian analysis, information on the choice of priors and Markov chain Monte Carlo settings
✓	☒	For hierarchical and complex designs, identification of the appropriate level for tests and full reporting of outcomes
✓	☒	Estimates of effect sizes (e.g. Cohen's *d*, Pearson's *r*), indicating how they were calculated

Our web collection on statistics for biologists contains articles on many of the points above.

Software and code

Policy information about availability of computer code

Data collection	For data collection, we used an open source smFRET software package developed by Taekjip Ha group, which is available from the group's website.
Data analysis	For data analysis, we used an open source smFRET software package developed by Taekjip Ha group, which is available from the group's website.

For manuscripts utilizing custom algorithms or software that are central to the research but not yet described in published literature, software must be made available to editors/reviewers. We strongly encourage code deposition in a community repository (e.g. GitHub). See the Nature Research guidelines for submitting code & software for further information.

Data

Policy information about availability of data

All manuscripts must include a data availability statement. This statement should provide the following information, where applicable:

- Accession codes, unique identifiers, or web links for publicly available datasets
- A list of figures that have associated raw data
- A description of any restrictions on data availability

(Online Methods; page 27 line 15) The datasets generated and/or analyzed during the current study are available from the corresponding author upon request.

Field-specific reporting

Please select the one below that is the best fit for your research. If you are not sure, read the appropriate sections before making your selection.

☒ Life sciences ☐ Behavioural & social sciences ☐ Ecological, evolutionary & environmental sciences

Life sciences study design

All studies must disclose on these points even when the disclosure is negative.

Sample size	For building each histogram in the paper, we collected > 30 movies which produce > 6000 single molecule spots, which is enough to build clear histograms, as can be seen from the small error ranges of the peak positions that are reported in supplementary tables.
Data exclusions	As a routine process in analyzing single molecule data, we excluded traces that do not contain a single pair of donor and acceptor dyes, which was judged by observing photobleaching steps and also checking acceptor fluorescence level upon acceptor excitation.
Replication	All the equilibrium measurements and non-equilibrium flow-in measurements were repeated measured by measuring multiple movies on the same day or repeating on another day and consistent behaviors were observed. The presented results include data analyzed from all measured movies.
Randomization	As we did not use organisms or participants, there was no need for randomization. Single molecule data analysis inherent randomize the observed molecules as we analyze all collected traces without discriminating against certain groups of samples.
Blinding	Group allocation was not necessary or relevant in this study.

Reporting for specific materials, systems and methods

We require information from authors about some types of materials, experimental systems and methods used in many studies. Here, indicate whether each material, system or method listed is relevant to your study. If you are not sure if a list item applies to your research, read the appropriate section before selecting a response.

Materials & experimental systems

n/a	Involved in the study
☒	☐ Antibodies
☒	☐ Eukaryotic cell lines
☒	☐ Palaeontology
☒	☐ Animals and other organisms
☒	☐ Human research participants
☒	☐ Clinical data

Methods

n/a	Involved in the study
☒	☐ ChIP-seq
☒	☐ Flow cytometry
☒	☐ MRI-based neuroimaging

Sample size: We added the following statement in revised Methods section to note the criteria of selecting sample sizes.
Each FRET histogram was built from > 50 movies, unless otherwise noted, that contain > 6,000 traces with a single pair of Cy3 and Cy5 dyes, which is enough to locate the center position of each FRET peak with accuracy < 0.03. Each FRET value in a histogram was represented by *E*FRET averaged over five frames.

Data exclusion: We excluded the traces from molecules that did not contain Cy5, because without Cy5, the molecule will not report any information on FRET efficiency. We revised Methods section as following to explain this criteria.
The acceptor dyes were briefly excited at the beginning and end of each movie and this was used to exclude traces lacking acceptor dyes from further analysis as the molecules without acceptor dyes do not report FRET efficiency.

Replication: We added the following statement in revised Methods section.
All measurements were repeated > 3 times and consistent behaviors were observed.

Randomization & Blinding: We added the following statement in revised Methods section, as suggested by the editor. Using no organisms or participants and including all collected traces for analysis make a common rationale for making no randomization and blinding control groups.
As we did not use organisms or participants, there was no need for randomization. Single molecule data analysis inherently randomizes the observed molecules as we analyze all collected traces without discriminating against certain groups of samples. For the same reason, group allocation for blinding was not necessary or relevant in this study.
